# Evaluation of diversity characteristics in a large mental healthcare data platform and their use in research publications: a cross-sectional review

**DOI:** 10.1136/bmjopen-2026-124809

**Published:** 2026-09-28

**Authors:** Alice Broadbent, Hannah Woods, Matthew Broadbent, Robert Stewart, Mariana Pinto da Costa

**Affiliations:** 1 Institute of Psychiatry, Psychology & Neuroscience, King's College London, London, UK; 2 South London and Maudsley NHS Foundation Trust, London, UK

**Keywords:** Health informatics, Research Design, Health

## Abstract

**Abstract:**

**Background:**

Reporting diversity characteristics is required for more inclusive, equitable and policy-relevant research.

**Objectives:**

To evaluate the reporting of diversity characteristics in publications using a large mental healthcare electronic health record (EHR)-derived research data resource, and to compare reporting of these characteristics in publications with their availabilities in the underlying dataset.

**Design:**

Cross-sectional review of Clinical Record Interactive Search (CRIS)-derived publications and assessment of diversity characteristic availability within the underlying EHR-derived database.

**Setting:**

The South London and Maudsley (SLaM) National Health Service (NHS) Foundation Trust Biomedical Research Centre Case Register, accessed via the CRIS platform, representing secondary mental healthcare delivered to a geographic catchment area covering four boroughs in south London.

**Methods and analysis:**

All CRIS-derived publications were reviewed to ascertain reporting of protected characteristics, as defined in the UK Equality Act 2010, alongside additional diversity-related characteristics. The availability of each characteristic within CRIS was assessed from records available up to 15 April 2026. Descriptive statistics were used to summarise reporting and data availability.

**Results:**

A total of 362 publications were evaluated. The mean number of diversity characteristics reported per publication was 4.0, and no publication reported more than 10 characteristics. Age (89.3%), sex (87.3%) and ethnicity (80.4%) were the most frequently reported characteristics. Socioeconomic status was reported in 42.5% of publications, while marriage and civil partnership (35.0%) and disability (27.0%) were reported in a smaller proportion of studies. All remaining characteristics were reported in less than 10% of publications.

Data availability within CRIS was highest for sex (99.9%) and age (99.8%), followed by socioeconomic status, geographic location and homelessness (all 97.5%) and ethnicity (86.5%). However, several characteristics were reported far less frequently than they were available in the dataset, particularly geographic location (6.6% reported despite 97.5% availability) and homelessness (5.8% reported despite 97.5% availability).

**Conclusions:**

Reporting of diversity characteristics in this case study for EHR-based mental health research was uneven and did not fully reflect availability in the source data. While age, sex and ethnicity are commonly reported, several other protected and diversity-related characteristics are rarely used by researchers despite their availability, and other characteristics remain challenging to capture. As routine EHR-derived datasets increasingly inform mental health research and policy, greater attention to recording, accessibility and reporting of diversity characteristics is required to support more inclusive and representative research.

**STRENGTHS AND LIMITATIONS OF THIS STUDY**

- Availability of diversity characteristics was assessed using predefined and consistently applied criteria across all variables.
- Reporting was evaluated through a review of Clinical Record Interactive Search-derived publications using a standardised data extraction approach.
- Findings are based on a single mental health electronic health record platform and may not generalise to all healthcare settings

## Introduction

 Mental health conditions affect individuals across all demographic groups. As a result, there is growing recognition of the need to ensure that research is representative of the population as a whole. Collecting and reporting diversity characteristics not only strengthens the validity of research findings but also helps to address healthcare disparities and promote more inclusive mental health services.^1–3^ The importance of diversity data has been emphasised by several national and international organisations, including the National Institute for Health and Care Research (NIHR) in the United Kingdom. The NIHR *Research Inclusion Strategy 2022–2027* recommends the recording of the protected characteristics in the UK Equality Act 2010 (age, disability, gender reassignment, marriage or civil partnership, pregnancy and maternity, ethnicity, religion or belief, sex and sexual orientation), as well as other factors, including socioeconomic status, geographic location and access to health and care.^4^ These recommendations reflect a growing expectation that routinely collected data should support inclusive, equitable and policy-relevant research.

Although previous research has examined the importance of diversity data, the main focus has been on participant recruitment.^5^ There remains a significant gap in the literature regarding the reporting of diversity data over time, particularly using the growing data resources derived from electronic health records (EHRs). In particular, there is limited evidence on how the availability of demographic data within EHR systems translates into their reporting in published research outputs, or how features within EHR systems, such as default values, conventions for missing data and the balance between structured fields and free-text documentation, influence research practices over time. The Clinical Record Interactive Search (CRIS) platform is an EHR-derived research data resource developed at the South London and Maudsley (SLaM) NHS Foundation Trust, a mental health NHS Trust serving four London boroughs: Croydon, Lambeth, Lewisham and Southwark. Reflecting the notably diverse population of south London compared with the wider UK, CRIS contains a very rich representation of different demographic groups. The Maudsley CRIS is among the most productive data resources in terms of mental health research outputs, across a wide range of topics^6^ with over 300 original research publications in peer-reviewed journals to date.

This study examined the extent to which diversity characteristics have been reported in publications using routinely collected mental healthcare data and compared this with the availability of the same characteristics in the source database. We assessed the reporting of the nine protected characteristics defined in the Equality Act 2010, alongside additional diversity-related variables, to identify gaps between what is recorded in routine clinical data and what is subsequently used and reported in published research. Understanding these discrepancies is important as EHR-derived datasets are increasingly used to inform the mental health evidence base, and could help guide improvements in the collection, accessibility and reporting of diversity data to support more inclusive and representative research. Equally, failure to represent diversity characteristics in EHR-derived research may inadvertently perpetuate, and potentially widen, existing inequalities in evidence.

## Methods

This study used a descriptive observational design to compare the availability of diversity characteristics within a large EHR-derived mental health research database with the reporting of these characteristics in published research outputs using the same data resource.

### Source of the data

SLaM developed a bespoke single EHR system—the electronic Patient Journey System—which was adopted across all its services in 2006. SLaM provides comprehensive mental healthcare to four diverse South London boroughs (Lambeth, Lewisham, Southwark and Croydon), with a combined population of approximately 1.3 million, as well as providing several national services.

CRIS was developed in 2007–2008 with funding from the NIHR and forms the platform for the SLaM BRC case register. CRIS structures and de-identifies complete information from the EHR for research use within a robust data securing and governance framework, with ethical approval for secondary analysis (Oxford REC C, 23/SC/0257) monitored by a patient-chaired Oversight Committee. Natural language processing (NLP) applications have been developed and widely deployed over a 10-year period to allow constructs of interest (eg, symptoms, risk factors and treatments) to be ascertained from free-text sections of the EHR, such as assessments, letters and other clinical documents.^7^ The SLaM Clinical Data Linkage Service was also established to enable CRIS data to be linked with other external data resources within a Trusted Research Environment. The CRIS platform enables researchers to access bespoke de-identified EHR-derived datasets, prepared by the CRIS Extraction Team, for Oversight Committee approved studies.

In line with the NIHR research inclusion strategy, this study focused on: (1) the nine protected characteristics defined in the Equality Act 2010—age, sex, ethnicity, marriage and civil partnership, disability, pregnancy and maternity, religion, sexual orientation and gender reassignment; (2) additional diversity data characteristics comprising socioeconomic status, employment status, homelessness, education status, geographic location, born outside the UK and migration status.

### Eligibility criteria

All publications that extracted data from CRIS were included. Publications such as meta-analyses or reviews of CRIS publications, abstracts only or commentaries on healthcare databases that did not extract new CRIS data were excluded. Publications were identified through a combination of the CRIS website publication list and an internally maintained compilation.

### Analysis methodology

Each included publication was independently reviewed by two researchers (AB and HW) to determine whether it reported one or more of the diversity characteristics of interest (see online supplemental table 1). Discrepancies between the two researchers were discussed and resolved through consultation with a third researcher (MPC). Reporting could be based on structured CRIS fields, NLP-derived variables or linked data sources.

The second stage involved assessing data availability within CRIS. All patient records available in CRIS as of 15 April 2026 were extracted. Availability was defined as the presence of a non-null, non-default recorded value considered meaningful for research according to predefined criteria for each diversity characteristic (see online supplemental table 2). These criteria varied by characteristic and could include structured EHR fields or NLP-derived variables where appropriate. For example, a valid date of birth (truncated for de-identification purposes to month and year of birth) was required for age, and a non-null value was required for ethnicity.

Where fields were auto-populated if missing, these variables were excluded. In addition, some diversity characteristics may be recorded primarily in free-text clinical documentation rather than structured fields. Where available, CRIS-derived variables generated through NLP were used. These factors were considered when defining meaningful values and when interpreting completeness estimates. As this definition is based on recorded values, availability does not necessarily equate to true data completeness and may overestimate true data quality, since systematic entry of default codes can satisfy the completeness threshold without reflecting real information.

### Data analysis

Descriptive statistics were used. Categorical and binary data were summarised as frequencies and proportions. Reporting of diversity characteristics was also summarised across four publication periods (2009–2012, 2013–2016, 2017–2020 and 2021–2025) to describe changes in reporting over time (figure 1). A year-by-year breakdown of reporting for each characteristic provided in online supplemental able 4.

**Figure 1:**
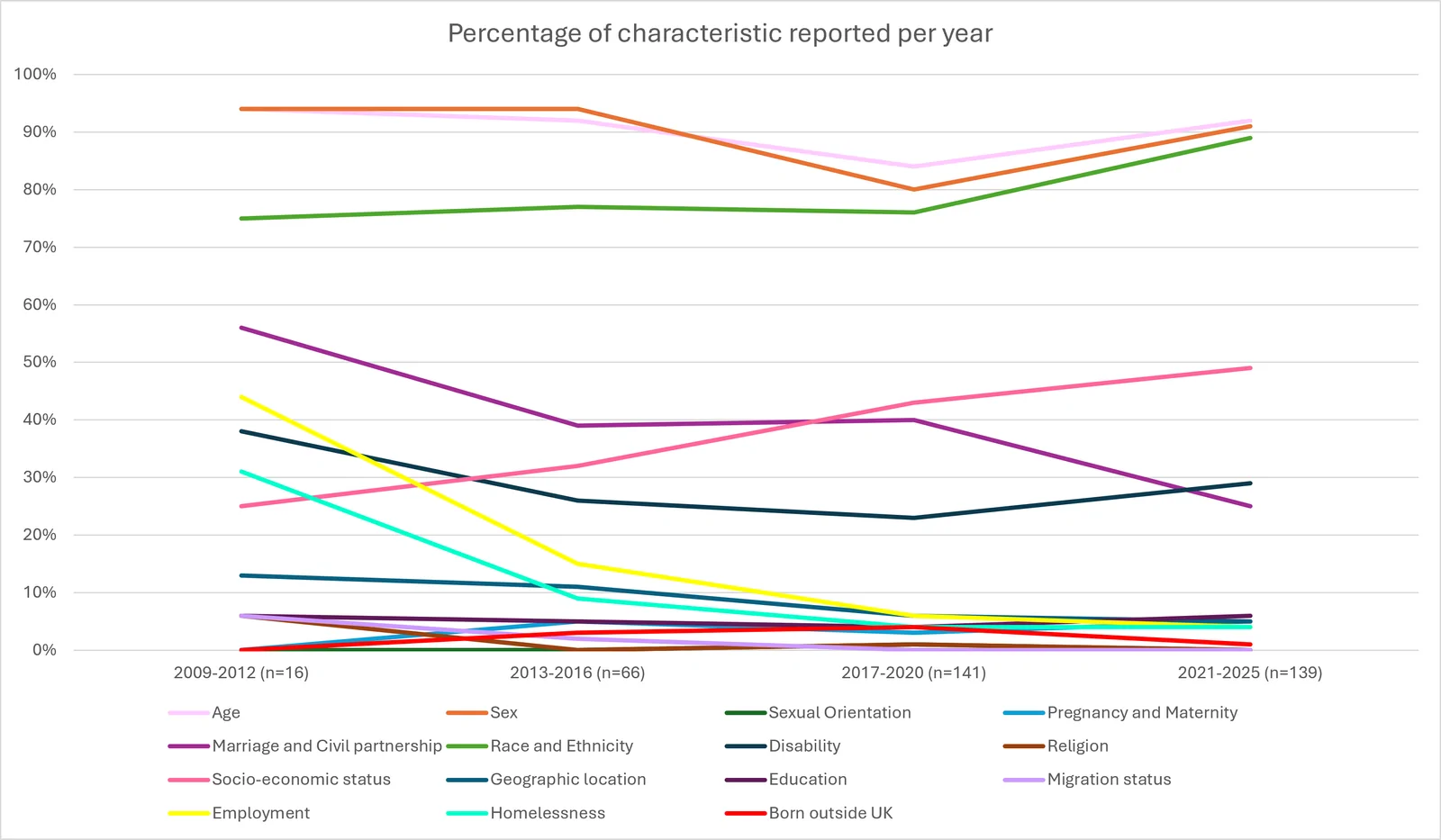
Percentage of publications reporting each diversity characteristic across publication periods, 2009–2025.

### Patient and public involvement

The CRIS platform, used in this study, is governed by a patient-led Oversight Committee, providing an indirect form of lay governance over the use of data. Additionally, the Data Linkage Service User and Carer Advisory Group advises on research using Data Linkages and the Clinical Informatics Service group advises on research using NLP.

## Results

A total of 411 publications were identified through the CRIS publication list. Following screening, 49 publications were excluded as they did not involve extraction of new CRIS data or did not meet the inclusion criteria. In total, 362 publications were included in the final analysis (figure 2). Of these, 144 (39.8%) reported using one or more linked data sources to derive additional information.

**Figure 2:**
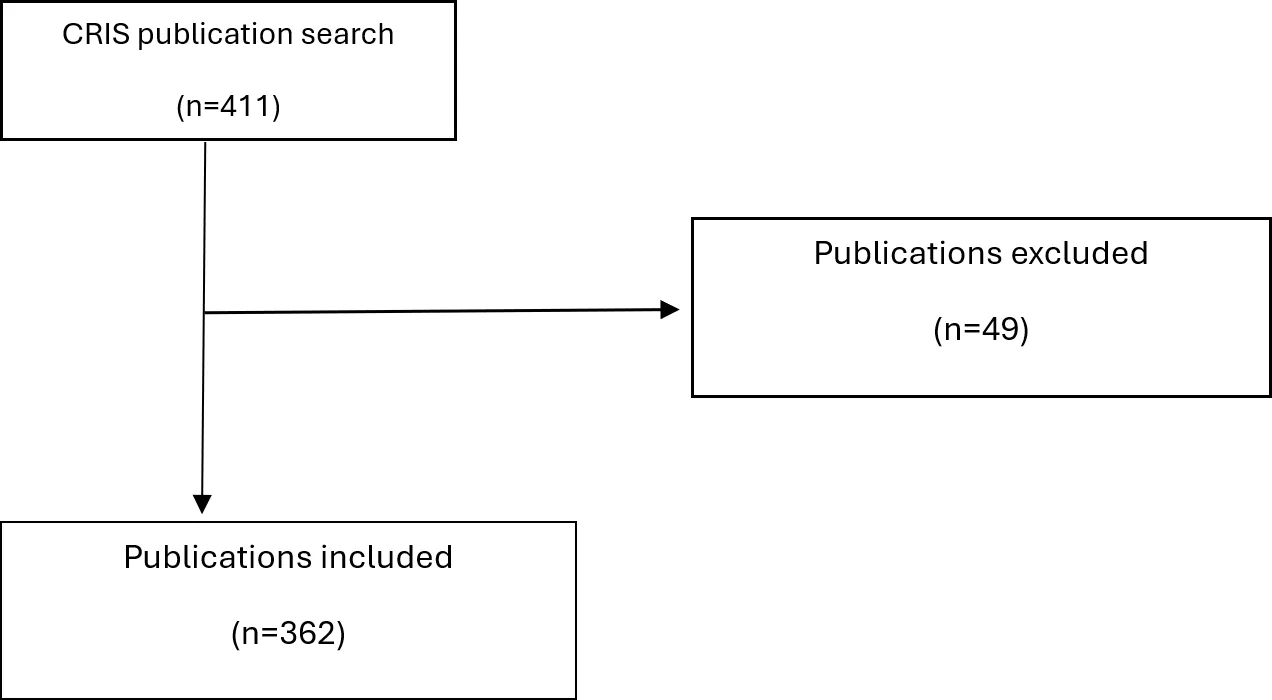
Publication review flow diagram. CRIS, Clinical Record Interactive Search.

Figure 3 summarises the number of protected and additional diversity characteristics reported in each publication. The mean number of characteristics reported per publication was 4.0 (SD=1.98), with the most common value being four characteristics (21.82% of papers). No publication reported more than ten characteristics.

**Figure 3:**
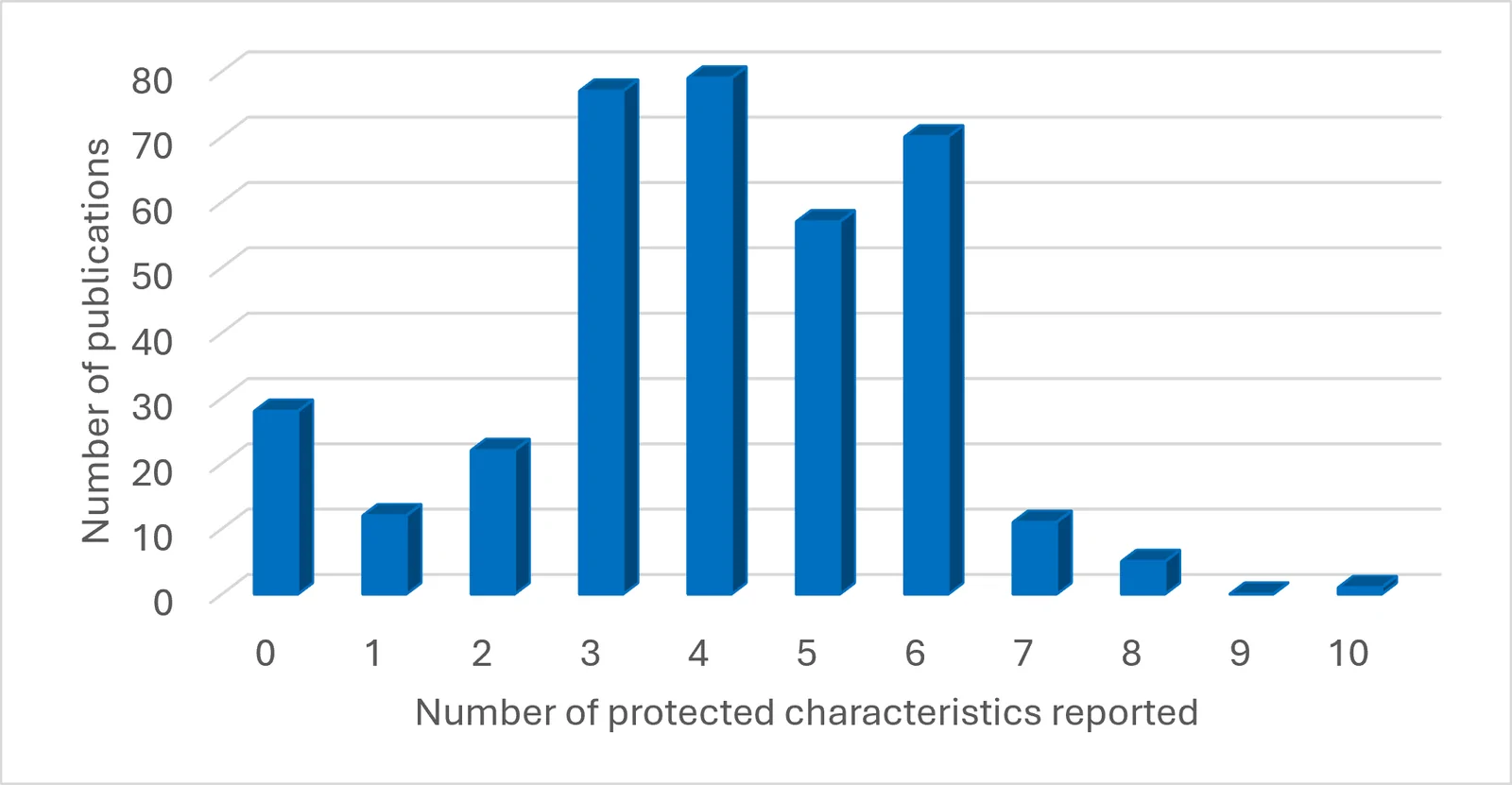
Distribution of number of protected and diversity characteristics reported by each publication.

The proportions of publications reporting each diversity characteristic are shown in figure 4. Age was the most frequently reported characteristic, included in 89.3% of publications, followed by sex (87.3%) and ethnicity (80.4%). Socioeconomic status was reported in fewer than half of publications, while marriage and civil partnership and disability were also reported in a minority of studies. All other characteristics were reported in fewer than 10% of publications.

**Figure 4:**
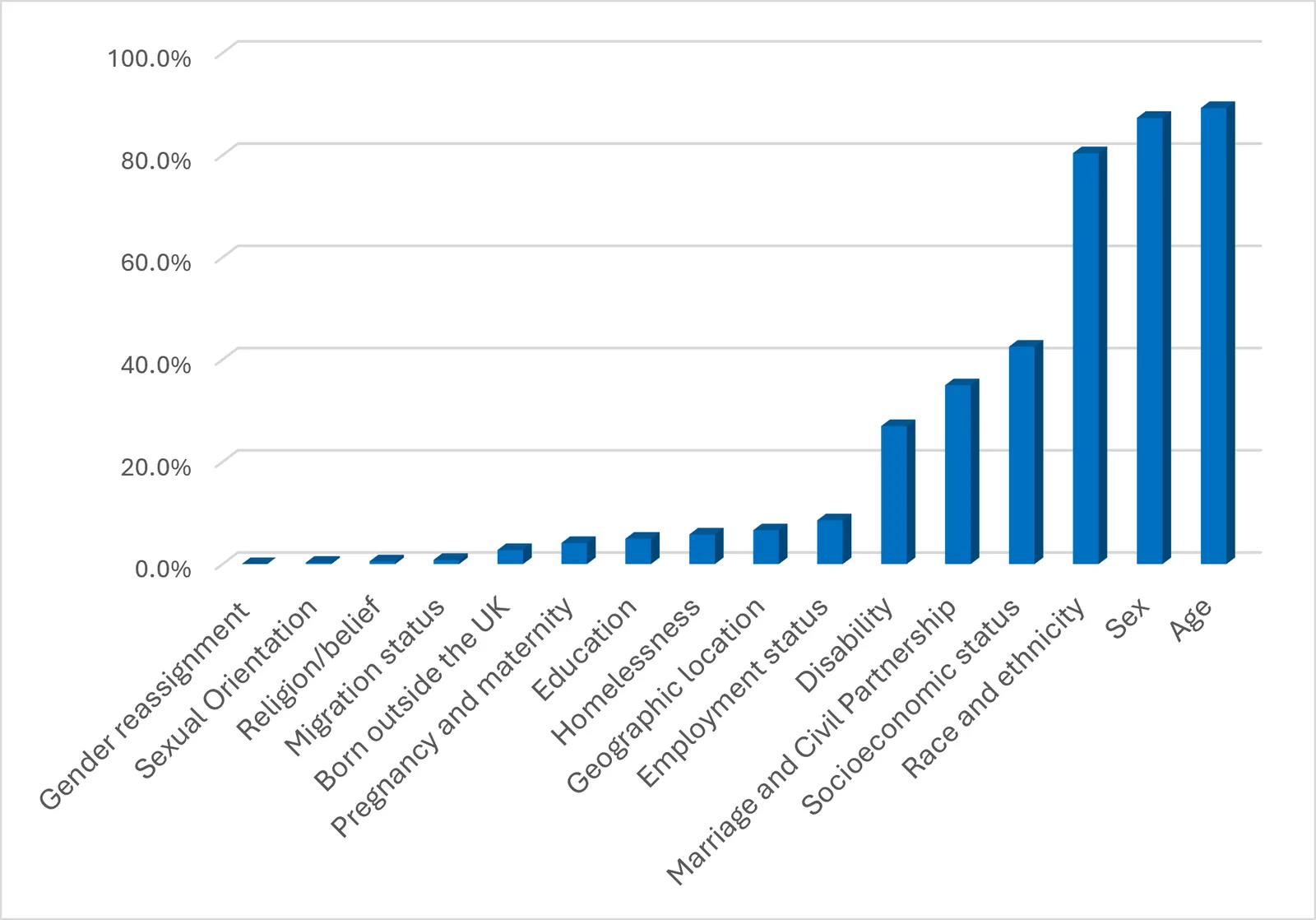
Percentage of publications reporting each characteristic.

Figure 5 compares the proportion of publications reporting each diversity characteristic with the availability of that characteristic in CRIS records. Age and sex showed both high availability and high reporting. Ethnicity was also widely available (86.5%) and frequently reported (80.4%).

**Figure 5:**
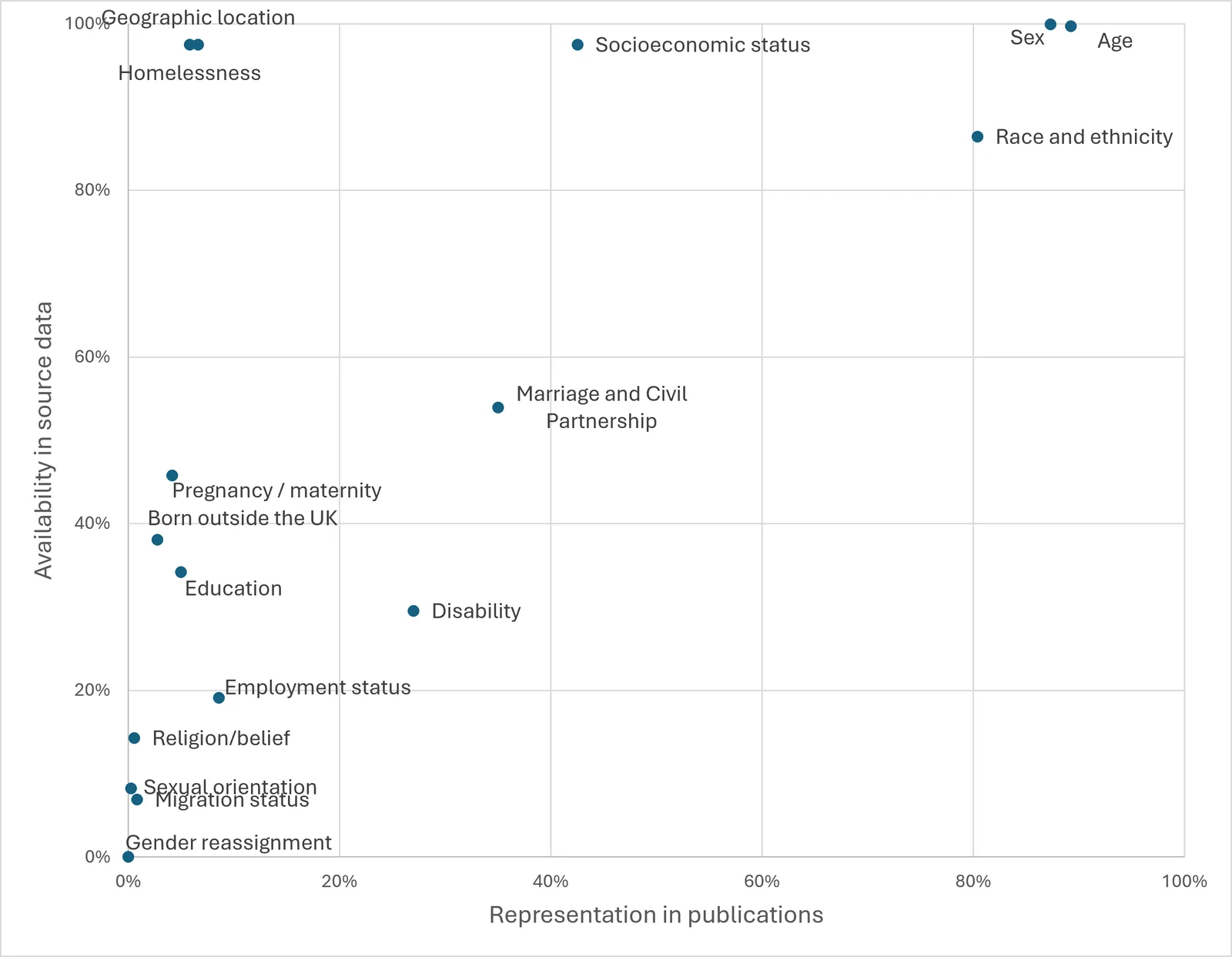
Scatterplot relationship between protected/diversity characteristic availability in source data and representation in publications.

Several characteristics showed substantial discrepancies between availability and reporting. For example, geographic location and homelessness were highly available in the source system (both 97.5%) but were reported in relatively few publications (6.6% and 5.8% respectively). Socioeconomic status was also highly available (97.5%) but was reported in only 42.5% of publications. Pregnancy/maternity showed moderate availability but was reported in only 4.12% of publications.

Other characteristics showed low availability and lower reporting, including religion (14.3% available, 0.55% reported), and migration status (6.9% available, 0.8% reported).

Overall, the availability within the underlying dataset did not consistently translate into reporting in publications. While characteristics such as age and sex were both widely available and consistently reported, many other characteristics were underreported relative to their potential availability, limiting the extent to which EHR-driven mental health research can describe and analyse outcomes across diverse groups.

## Discussion

In a large EHR-derived data resource widely used to support mental health research over a long period, we evaluated the representation of diversity characteristics in published output to date and the extent to which this has reflected availability in the source data. The most frequently reported diversity characteristic in publications was age (89.2%), followed by sex (87.3%), and these two characteristics also had the highest availability in the CRIS database (age: 99.8%, sex: 99.9%). Ethnicity was also widely available (86.48%) and frequently reported (80.39%). Other diversity-related characteristics were less commonly reported. This pattern did not always reflect data availability, with some characteristics showing low reporting despite being recorded for a proportion of patients, although others more clearly reflected limited or no availability within the source EHR-derived data. These findings suggest that the representation of diversity characteristics in EHR-based mental health research is shaped not only by what is recorded in routine care but also by what researchers choose to extract, analyse and report.

A key finding of this study is that availability of diversity characteristics within the dataset did not consistently translate into their use or reporting in publications. While age and sex were both frequently reported, several other characteristics were infrequently reported even when data were available.

Several factors may help explain this gap. Researchers may be uncertain about the validity or completeness of certain variables, particularly where measurement is inconsistent or not well standardised. In some cases, the way variables are recorded also limits their usefulness. For example, the temporality of certain characteristics can raise concerns about validity when only a single time-point value is available for concepts that may change over time, such as marital status. This may discourage researchers from using such information if it is perceived as unrepresentative or unreliable. Additionally, researchers may prioritise variables that are already widely used in the literature and expected by journals or reviewers, such as age, sex and ethnicity, while other characteristics may be overlooked despite being relevant to understanding inequalities in mental health outcomes.

Notably, several characteristics showed high availability in CRIS but were rarely reported in publications. For example, geographic location and homelessness were recorded for almost all patients (both 97.52%) but were reported in fewer than 10% of publications. Socioeconomic status was also highly available (97.51%) but was reported in fewer than half of publications. Pregnancy and maternity showed moderate availability (46%) but were reported in only 4.14% of publications.

The risk is that such cumulative gaps in the use of diversity-related information may become self-reinforcing and contribute to an impoverished evidence base. For example, if marital status is not well completed in source records, the evidence base on its influence on mental health outcomes is less well understood. This, in turn, may lead to an underestimation of its importance in healthcare policy, further diminishing the motivation for its systematic recording in EHRs.

The discrepancies observed in this study suggest that underuse of certain characteristics may not solely reflect incomplete recording, but may also reflect selective use of available variables in research practice.

It is also important to note that availability in routinely collected EHR-derived datasets does not necessarily equate to true completeness or usability. The recording of diversity characteristics in routine care may be influenced by clinical workflows, staff training, perceived relevance and the sensitivity of certain topics.

The availability of different variables in the source system depends on the reasons for which they are recorded. Variables may be captured for clinical purposes (eg, sex), administrative requirements (eg, age), or performance management incentives (eg, ethnicity). While administratively relevant information is typically recorded in structured fields, clinically relevant information is often documented within unstructured clinical text. As a result, the absence of a structured entry does not necessarily indicate that the characteristic is not recorded elsewhere in the patient record.

Although systems such as CRIS allow users to search free text and identify mentions of specific entities within clinical notes, research use of this information at scale largely depends on NLP applications. This highlights the importance of NLP approaches in improving the accessibility of characteristics that are not consistently captured in structured formats. For example, a recent NLP application developed to ascertain LGBTQ+status^8^ demonstrates how such methods can expand opportunities to investigate intervention and outcome equity in previously underexplored populations.

Concerns regarding the representation and reporting of diversity characteristics are well established in clinical trials research, where the under-representation of minority groups and inconsistent reporting of participant characteristics can limit generalisability.^9 10^ However, the factors influencing the representation and reporting of diversity characteristics may differ across study designs. In trials, under-representation is often driven by recruitment processes and eligibility criteria, whereas in EHR-based research, inclusivity is theoretically complete (due to universal healthcare provision and representation in the source EHR). However, its visibility and utility for research are influenced by the availability and quality of routine clinical recording, as well as by the decisions made during data extraction and analysis. The limited literature examining diversity reporting in routine EHR-based research suggests that this matter has received less attention than recruitment-based inequalities in trials, despite the increasing use of routinely collected data in mental health research and policy.^3 5^

These findings have several implications for improving diversity and inclusivity in routine-data research. First, clinical services and healthcare organisations may need to place greater emphasis on the consistent recording of key equality and diversity characteristics. However, this should be accompanied by meaningful incentives and clear communication to ensure that data entry is both accurate and complete.

For example, in 2023 the NHS Trust made completion of the ethnicity field in the patient demographic form mandatory, which resulted in reported completeness reaching 100% in subsequent years. However, closer examination of the data suggests that the category ‘z-code not stated’ is frequently selected (see online supplemental table 3), indicating that staff may use this option as a default when a patient’s ethnicity is unknown. This creates the appearance of full completeness while potentially obscuring true data quality.

In contrast, the ‘Child Need and Risk’ form, used to derive the maternity variable and also mandatory, appears to be filled out more completely.^11^ It is important to note that this form is only completed for adult patients and teenagers who are pregnant; therefore, estimates of availability for this variable may be misleadingly low in datasets that include Child and Adolescent Mental Health Services (CAMHS) and older adult populations, for whom the form is not routinely used. Approximately 2/3 of SLaM service users are working age adults.^6^ However, the majority of maternity and pregnancy-related research uses the linkage between CRIS and Hospital Episode Statistics to ascertain delivery episodes rather than this form.

Overall, these findings suggest that performance management approaches alone are insufficient. Where such measures are introduced, they must be accompanied by appropriate staff training and a clear understanding of why accurate recording of specific variables is important, to avoid the creation of misleading indicators of data completeness.

While some improvement in information might be achieved by additional structured fields, these tend to be unpopular with clinical practitioners and sustainability of high levels of completion has yet to be demonstrated. Research funders and policy-makers should therefore support the development of infrastructure that enables better use of routinely collected diversity data, making better use of data already collected rather than requiring more ‘box-ticking’ in the EHR. Additionally, research funders and journal publishers could require more consistent reporting of diversity characteristics by requiring or incentivising reporting of protected characteristics as part of submission checklists or grant reviews.

Most information in mental health records is contained in free text entries rather than structured fields, which limits its accessibility for analysis. However, NLP applications have been demonstrated as feasible for extracting specific entities within unstructured clinical notes. As of June 2026, there are over 100 NLP applications within CRIS^7^ and these have not only underpinned most research output from CRIS to date, but are also now deployed in individual and caseload-/service-level visualisations at the EHR interface via SLaM’s Visual & Interactive Engagement With Electronic Records (VIEWER) prototype and Locating Useful Clinical Information service.^12 13^

Linkages to external datasets may improve the ascertainment of characteristics that are not consistently captured in routine mental health notes. As of June 2026, CRIS is linked with 12 external datasets.^14^ However, external data linkages are primarily of use for research rather than service surveillance, as they are often either one-off assemblies of data or, when updated, contain information at least a year old, limiting their real-time utility.

The findings of this study highlight the need for greater focus on the equity implications of routine data systems. As EHR-derived datasets become a major source of evidence, the completeness and usability of diversity characteristics within these datasets become increasingly important. Without improvements, there is a risk that research based on routine data will continue to disproportionately represent groups for whom information is more consistently recorded, while excluding others.^2^

### Strengths and limitations

To our knowledge, this article reports the first case study to systematically examine the reporting of diversity data in publications derived from a large health records database. It provides a comprehensive assessment of diversity reporting across a substantial number of publications derived from a major mental health EHR research platform, and offers a direct comparison between publication reporting and underlying data availability, allowing identification of systematic discrepancies.

However, some limitations should be considered. First, this analysis focused on one research platform and therefore may not fully generalise to other health systems. Nevertheless, many of the underlying challenges relating to routine recording practices and missingness are likely to apply across settings and we hope that the approach taken here provides a model for wider investigation and collation of experiences. Second, availability was assessed based on the presence of recorded values. Third, publications may use diversity variables in analyses without explicitly reporting them in full, meaning that reported figures may underestimate their actual use. Importantly, this bias is directional: the reported figures should be interpreted as conservative lower bounds, and the true gap between the availability and use of diversity characteristics may be smaller than the data suggest, particularly for characteristics such as socioeconomic status or geographic location, which may appear implicitly in sub-group analyses without being named. Finally, some characteristics may be available through linkages or extraction methods not consistently described in publications, which may have affected classification. In addition, it should be noted that gender reassignment data were not available in CRIS at the time of extraction, although a gender identity field was introduced in the source EHR in 2025.

## Conclusions

This study demonstrates that the reporting of diversity characteristics in EHR-based mental health research is not fully explained by the availability of data in the underlying dataset. While age, sex and ethnicity are widely used, many other protected and diversity-related characteristics are rarely reported, limiting the ability of routine-data research to address inequalities across the full range of demographic groups. As routine clinical datasets increasingly inform mental health evidence and policy, greater attention to the availability, usability and reporting of diversity characteristics is needed. Improving inclusivity will require coordinated efforts across clinical recording practices, research infrastructure and reporting standards.

## Supplementary material

10.1136/bmjopen-2026-124809 online supplemental file 1

## Data Availability

Data are available upon reasonable request.
