# Supplementary material for: Evaluation of diversity characteristics in a large mental healthcare data platform and their use in research publications: a cross-sectional review
Source: BMJ Open. 2026 Sep 28;16(9):e124809. doi: 10.1136/bmjopen-2026-124809 (PMC13630100; doi:10.1136/bmjopen-2026-124809)
Supplement: Supplementary file 1 [file bmjopen-2026-124809.reviewer_comments.pdf]

PEER REVIEW HISTORY

BMJ Open publishes all reviews undertaken for accepted manuscripts. Reviewers are asked to complete a checklist review form and are provided with free text boxes to elaborate on their assessment. These free text comments are reproduced below.

ARTICLE DETAILS

Title (Provisional)

An evaluation of diversity characteristics in a large mental healthcare data platform and their use in research publications: a cross-sectional review.

Authors

Broadbent, Alice; Woods, Hannah; Broadbent, Matthew; Stewart, Robert; Pinto da Costa, Mariana

---

VERSION 1 - REVIEW

Reviewer 1

Name Găină, Marcel

Affiliation Grigore T Popa University of Medicine and Pharmacy Faculty of Medicine, 3rd Medical, Psychiatry

Date 19-Jul-2026

COI None

Esteemed Authors of manuscript: An evaluation of diversity characteristics in a large mental healthcare data platform and their use in research publications

Allow me to begin by expressing my genuine appreciation for the work you have submitted; I am lucky as a peer review to encounter such valuable work. What you have built here is something the field has quietly needed for a long time: a rigorous, transparent audit that looks beyond the data itself and asks a harder question — are we actually using what we have, and are we doing so in a way that serves everyone equally? The fact that this audit is grounded in one of the most productive mental health EHR platforms in the world (-CRIS, South London and Maudsley NHS Foundation Trust) gives it immediate credibility and real-world weight that purely theoretical discussions about diversity reporting simply cannot match.

The scatterplot in Figure 4 alone tells a story that no amount of narrative prose could replace: geographic location and homelessness recorded in 97.5% of patient records, yet reported in only 6.6% and 5.8% of publications respectively. That gap is not a methodological artefact — it is a reflection of how choices made at the desk, not at the bedside, quietly shape whose lives appear in the evidence base. Your framing of this as a potentially self-

reinforcing cycle (page 8–9) is one of the most honest and important observations in the paper.

Please receive these following advisory notes only where you feel they preserve and strengthen your own voice and argument. I look forward to seeing this paper published, so that I may cite it in my own work.

Advisory notes

1.Clarify the definition of "availability" more explicitly upfront

Where: Page 5–6, Methods (Source of the data; Analysis methodology); Supplementary Tables 1 and 2.

What is needed: I belive that the distinction between a variable being recorded in CRIS and being meaningfully usable for research is well handled in the supplementary material, but it appears rather late for the general reader. I would advise perhaps adding one to three sentences in the Methods section (page 5–6) that explicitly state: 1st availability was defined as the presence of a non-null, non-default, meaningful value in the structured field or NLP-derived output; 2nd this definition may still overestimate true data quality, since systematic entry of default codes (e.g., "z-code not stated" for ethnicity, Supplementary Table 3) can satisfy the completeness threshold without reflecting real information.

Why this matters: Without this anchor early in the paper, the reader arrives at the Discussion's nuanced ethnicity example (page 9–10) — where 100% completeness in 2025 co-exists with 23% z-code usage — without a conceptual framework. This finding is one of your strongest real-world contributions; it deserves to be set up, not discovered mid-Discussion.

2.Clarify the directionality of the reporting underestimation bias

Where: Page 11, Limitations, lines 11–14.

What is needed: You correctly note that "publications may use diversity variables in analysis without explicitly reporting them in full, meaning that reporting may underestimate actual usage." I would advise one additional sentence acknowledging that this bias is directional: your reported figures are conservative lower bounds, meaning the true gap between availability and use may be smaller than the data suggest — or differently structured — particularly for characteristics such as socioeconomic status or geographic location, which may appear implicitly in sub-group analyses without being named.

Why this matters: Without this clarification, readers may treat your reporting percentages as exact estimates rather than lower bounds, which risks overstating the severity of the gap for some characteristics.

3.Strengthen the link to actionable reporting standards

Where: Page 10–11, Discussion, implications paragraph.

What is needed: Your distinction between performance management that creates misleading completeness versus meaningful incentives backed by training is genuinely important (page 10). I would advise making the policy pathway more explicit: a single sentence noting that journal editors and research funders — including NIHR, whose inclusion strategy you cite — could require or incentivise reporting of protected characteristics as part of submission checklists or grant reviews would give BMJ Open's readership (clinicians, researchers, policymakers) a concrete next step anchored in your findings.

Why this matters: Your data are compelling enough to support a recommendation. The absence of such recommendation exposes the Discussion section slightly open-ended, given the clarity of the problem documented. Still, this is NOT MANDATORY in the final perspective of this peer reviewer.

4.Consider to one sentence to the patient and public involvement section

Where: Page 6, Patient and Public Involvement.

What is needed: The transparent acknowledgement that patients were not involved in study design is appropriate. However, since the CRIS platform itself is governed by a patient-chaired Oversight Committee (mentioned page 5), I would advise one sentence acknowledging that this mechanism provides an indirect form of lay governance over the use of the data, even if formal PPI in study design was not conducted.

Why this matters: It is a small but meaningful clarification that positions the work correctly and avoids an unnecessary editorial query at the BMJ Open stage, ensuring acceptanvce.

5.Minor typographic issue

Where: Page 2, Abstract, Design section.

"Cross-sectional review of CRIS-derived publications and assessment of diversity characteristic availability within the underlying HER-derived database" — this should be EHR-derived instead, if I did not misunderstand the abbreviation properly, do let me know; otherwise, please verify before final submission. This is a typo, perhaps, still, do check it out.

These five notes are offered in the spirit of strengthening furthermore a paper that is already genuinely valuable. Please feel free to disagree with any of them; your authorial judgment takes precedence. I look forward with real interest to this paper's publication; also - please feel free to contradict me, and not agree with my perspective. I look forward seeing this paper published, so that I myself can cite it in my own works.

Yours truly grateful for the opportunity to review this valuable manuscript,

---

**Reviewer**	**2**
**Name**	**Jaisoorya, TS**
**Affiliation**	**NIMHANS, Psychiatry**

**Date**	**04-Aug-2026**
**COI**	**None**

The manuscript is well written and addresses an important aspect of EHR research. It can be considered for publication.

A few suggestions for consideration:

In the abstract, a brief background of 1–2 sentences (or the need for the study) placed before the objectives—for example: "Reporting diversity characteristics is required for more inclusive, equitable, and policy-relevant research"—will help contextualize the manuscript.

The key finding of this study is that the availability of diversity characteristics within a dataset did not consistently translate into their use or reporting in publications. However, in most case this disparity primarily relates to how relevant the authors considered these characteristics for inclusion, especially given that most journals enforce word counts and table size limits. However, I accept that most of these characteristics remain of primary importance in understanding inequalities in mental health outcomes.

Some exploratory subgroup analyses will help clarify the data:

• Among publications released after the advisory (the 2022 NIH inclusion advisory), what proportion reported these data?

• Among publications released after the implementation of NLP applications, has there been a shift in reporting patterns

---

VERSION 1 - AUTHOR RESPONSE

**Reviewer #1**		
Clarify the definition of "availability" more explicitly upfront<br><br>What is needed: I believe that the distinction between a variable being recorded in CRIS and being meaningfully usable for research is well handled in the supplementary material, but it appears rather late for the general reader. I would advise perhaps adding one to three sentences in the Methods section (page 5–6) that explicitly state:<br>1st availability was defined	We have now revised the text: “Availability was defined as the presence of a non-null, non-default recorded value considered meaningful for research according to predefined criteria for each diversity characteristic (supplementary table 2). These criteria varied by characteristic and could include structured EHR fields or NLP-derived variables where appropriate.<br>As this definition is based on recorded values, availability does not necessarily equate to true data completeness	Where: Page 5–6, Methods (Source of the data; Analysis methodology); Supplementary Tables 1 and 2.

as the presence of a non-null, non-default, meaningful value in the structured field or NLP-derived output;<br>2nd this definition may still overestimate true data quality, since systematic entry of default codes (e.g., "z-code not stated" for ethnicity, Supplementary Table 3) can satisfy the completeness threshold without reflecting real information.<br>Why this matters: Without this anchor early in the paper, the reader arrives at the Discussion's nuanced ethnicity example (page 9–10) — where 100% completeness in 2025 co-exists with 23% z-code usage — without a conceptual framework. This finding is one of your strongest real-world contributions; it deserves to be set up, not discovered mid-Discussion.	and may overestimate true data quality, since systematic entry of default codes can satisfy the completeness threshold without reflecting real information."	
Clarify the directionality of the reporting underestimation bias<br><br>What is needed: You correctly note that "publications may use diversity variables in analysis without explicitly reporting them in full, meaning that reporting may underestimate actual usage." I would advise one additional sentence acknowledging that this bias is directional: your reported figures are conservative lower bounds, meaning the true gap between availability and use may be smaller than the data suggest — or differently structured — particularly for	We have now revised the text: "Publications may use diversity variables in analyses without explicitly reporting them in full, meaning that reported figures may underestimate their actual use. Importantly, this bias is directional: the reported figures should be interpreted as conservative lower bounds, and the true gap between the availability and use of diversity characteristics may be smaller than the data suggest, particularly for characteristics such as socioeconomic status or geographic location, which may appear implicitly in sub-	Page 11, Limitations, lines 11–14.

characteristics such as socioeconomic status or geographic location, which may appear implicitly in sub-group analyses without being named.<br><br>Why this matters: Without this clarification, readers may treat your reporting percentages as exact estimates rather than lower bounds, which risks overstating the severity of the gap for some characteristics.	group analyses without being named."	
Strengthen the link to actionable reporting standards<br><br>What is needed: Your distinction between performance management that creates misleading completeness versus meaningful incentives backed by training is genuinely important (page 10). I would advise making the policy pathway more explicit: a single sentence noting that journal editors and research funders — including NIHR, whose inclusion strategy you cite — could require or incentivise reporting of protected characteristics as part of submission checklists or grant reviews would give BMJ Open's readership (clinicians, researchers, policymakers) a concrete next step anchored in your findings.<br>Why this matters: Your data are compelling enough to support a recommendation. The absence of such recommendation exposes the Discussion section slightly open-ended, given	We have now revised the text: "Research funders and journal editors could play an important role in encouraging more consistent reporting of diversity characteristics by requiring or incentivising reporting of protected characteristics as part of submission checklists or grant reviews."	Page 10–11, Discussion, implications paragraph.

the clarity of the problem documented. Still, this is NOT MANDATORY in the final perspective of this peer reviewer.		
Consider to one sentence to the patient and public involvement section<br><br>What is needed: The transparent acknowledgement that patients were not involved in study design is appropriate. However, since the CRIS platform itself is governed by a patient-chaired Oversight Committee (mentioned page 5), I would advise one sentence acknowledging that this mechanism provides an indirect form of lay governance over the use of the data, even if formal PPI in study design was not conducted.<br>Why this matters: It is a small but meaningful clarification that positions the work correctly and avoids an unnecessary editorial query at the BMJ Open stage, ensuring acceptance.	We have now added the sentence: "The CRIS platform, used in this study, is governed by a patient-led Oversight Committee, providing an indirect form of lay governance over the use of data. Additionally, the Data Linkage Service User and Carer Advisory Group (DL-SUCAG) advises on research using Data Linkages and the Clinical Informatics Service (CIS) group advises on research using NLP."	Page 6, Patient and Public Involvement.
Minor typographic issue<br><br>"Cross-sectional review of CRIS-derived publications and assessment of diversity characteristic availability within the underlying HER-derived database" — this should be EHR-derived instead, if I did not misunderstand the abbreviation properly, do let me know; otherwise, please verify before final submission. This is a typo, perhaps, still, do check it out.	Thank you. We have now corrected the typo. "Cross-sectional review of CRIS-derived publications and assessment of diversity characteristic availability within the underlying EHR-derived database"	Where: Page 2, Abstract, Design section.
**Reviewer #2**		

In the abstract, a brief background of 1–2 sentences (or the need for the study) placed before the objectives—for example: "Reporting diversity characteristics is required for more inclusive, equitable, and policy-relevant research"—will help contextualize the manuscript.	Thank You. We have now added the sentence: "Reporting diversity characteristics is required for more inclusive, equitable, and policy-relevant research"	Abstract - Objectives
The key finding of this study is that the availability of diversity characteristics within a dataset did not consistently translate into their use or reporting in publications. However, in most case this disparity primarily relates to how relevant the authors considered these characteristics for inclusion, especially given that most journals enforce word counts and table size limits.<br>However, I accept that most of these characteristics remain of primary importance in understanding inequalities in mental health outcomes.<br>Some exploratory subgroup analyses will help clarify the data:<br>• Among publications released after the advisory (the 2022 NIH inclusion advisory), what proportion reported these data?<br>• Among publications released after the implementation of NLP applications, has there been a shift in reporting patterns	Figure 5 shows reporting of diversity characteristics was also summarised across four publication periods (2009–2012, 2013–2016, 2017–2020 and 2021–2025) to describe changes in reporting over time. Table 4 in the supplementary document shows a year-by-year breakdown.<br><br>Education and LGBT have NLP applications. The Education NLP application was introduced in 2012, but as seen from the data has not had an impact on the number of times it is reported in publications.<br>The LGBT app was introduced in 2025 so our findings don't cover any changes it might make.	Results Figure 5
