# Supplementary material for: Evaluation of diversity characteristics in a large mental healthcare data platform and their use in research publications: a cross-sectional review
Source: BMJ Open. 2026 Sep 28;16(9):e124809. doi: 10.1136/bmjopen-2026-124809 (PMC13630100; doi:10.1136/bmjopen-2026-124809)
Supplement: Supplementary file 2 [file bmjopen-16-9-s001.docx]

| **Characteristic** | **Meaningful values (included in analysis)** | **Not meaningful values (excluded from analysis)** |
| --- | --- | --- |
| Age | Date of birth | N/A |
| Sex | Sex | Unknown |
| Ethnic group | Ethnic group, including "other ethnic group" | Not known; Not stated, Null |
| Socioeconomic status | Occupation; truncated address; welfare benefits; housing status (including "other"); employment status (including "other") | Occupation: Not applicable, Not known; Housing status: Not known, Null; Employment status: Not known, Null |
| Marriage and civil partnership | Marital status | Unknown |
| Disability | Mobility problems; hearing impairment; visual impairment; HoNoS data | Mobility problem: No known problem; Hearing impairment: No known problem, Not known; Visual impairment: No known problem, Not known |
| Migration status | Overseas visitor; Asylum seeker; Country of Origin | Asylum seeker: Null; Country of origin: Not stated, Null |
| Employment status | Employment status (including "other"); Occupation | Employment status: Not known, Null; Occupation; Not applicable; Not known |
| Homelessness | Housing status (Including "other"); Homeless indicator | Housing status: Not known, Null |
| Education | Education NLP application or linkage with National Pupil Database (NPD) | N/A |
| Geographic loaction |  | N/A |
| Born outside the UK | Country of origin | Not stated; Null |
| Religion or belief | Religion, including "any other religion" | Not known; Null; Religion not given |
| Sexual orientation | Sexual orientation including "other/not listed" and "not known" | Not stated, Null |
| Gender reassignment | Not avaliable in CRIS | N/A |
| Pregnancy and maternity | Hospital Episode Statistics (HES) linkage | N/A |

**Table S1: Definition of meaningful and not meaningful values for diversity characteristics**

**Table S2: Criteria for meaningful data in EPJS**

| **Variable** | **Meaningful** |
| --- | --- |
| Age | Cleaned date of birth from Patient Demographics form |
| Sex | Sex in Patient Demographics form |
| Socio-economic status | Address |
| Marriage and civil partnership | Marital status from Patient Demographics form |
| Disability | Disability in Patient Demographics form + HoNoS |
| Pregnancy and Maternity | Child need and risk form |
| Migration status | Asylum seeker |
| Employment status | Employment status from Patient Demographics form + summary of need form |
| Education | Education NLP application |
| Geographic Location | Address |
| Born outside the UK | Country of origin in Patient Demographics form |
| Religion or belief | Religion from Patient Demographics form |
| Sexual orientation | Sexual orientation in Patient Demographic form + sexuality NLP |
| Gender reassignment | Not available in CRIS |
| Homelessness | Address in Patients Demographics form |

**Table S3: Epjs ethnicity categories**

| **Year of registration** | **Number of new epjs registrations** | **Percentage with ethnicity** | **Percentage with z-code ethnicity** |
| --- | --- | --- | --- |
| 2010 | 19869 | 92 | 5 |
| 2011 | 19324 | 92 | 6 |
| 2012 | 19836 | 90 | 6 |
| 2013 | 19714 | 89 | 7 |
| 2014 | 20454 | 87 | 8 |
| 2015 | 22237 | 85 | 9 |
| 2016 | 22500 | 81 | 11 |
| 2017 | 23442 | 80 | 13 |
| 2018 | 24386 | 70 | 8 |
| 2019 | 26387 | 61 | 4 |
| 2020 | 23954 | 65 | 5 |
| 2021 | 26955 | 75 | 9 |
| 2022 | 29418 | 78 | 8 |
| 2023 | 28235 | 95 | 17 |
| 2024 | 28658 | 99 | 22 |
| 2025 | 29286 | 100 | 23 |

**Table S4: Percentage of publications reporting each diversity characteristic by publication year, 2009–2025.**

| **Year** | **Age** | **Sex** | **Sexual Orientation** | **Pregnancy and Maternity** | **Marriage and Civil Partnership** | **Race and Ethnicity** | **Disability** | **Religion** | **Socioeconomic Status** | **Geographic Location** | **Migration Status** | **Employment** | **Homelessness** | **Born outside the UK** |
| --- | --- | --- | --- | --- | --- | --- | --- | --- | --- | --- | --- | --- | --- | --- |
| 2009 n=1 | 100% | 100% | 0% | 0% | 0% | 100% | 0% | 0% | 100% | 0% | 100% | 100% | 0% | 0% |
| 2010 n=1 | 100% | 100% | 0% | 0% | 0% | 100% | 0% | 0% | 0% | 0% | 0% | 0% | 0% | 0% |
| 2011 n=5 | 80% | 80% | 0% | 0% | 40% | 60% | 20% | 20% | 0% | 20% | 0% | 20% | 20% | 0% |
| 2012 n=5 | 100% | 100% | 0% | 0% | 78% | 78% | 56% | 56% | 33% | 11% | 0% | 55% | 44% | 0% |
| 2013 n-7 | 86% | 71% | 0% | 0% | 29% | 43% | 29% | 29% | 14% | 14% | 0% | 0% | 14% | 0% |
| 2014 n=18 | 88% | 94% | 0% | 0% | 33% | 88% | 39% | 39% | 44% | 6% | 11% | 22% | 17% | 0% |
| 2015 n=16 | 93% | 93% | 0% | 13% | 53% | 73% | 13% | 13% | 13% | 13% | 0% | 20% | 0% | 0% |
| 2016 n=26 | 96% | 100% | 0% | 4% | 38% | 81% | 23% | 23% | 38% | 12% | 4% | 12% | 8% | 8% |
| 2017 n=24 | 79% | 79% | 0% | 0% | 33% | 66% | 15% | 15% | 21% | 0% | 8% | 4% | 0% | 13% |
| 2018 n=33 | 85% | 82% | 3% | 6% | 39% | 73% | 27% | 27% | 36% | 0% | 0% | 9% | 3% | 3% |
| 2019 n=35 | 83% | 80% | 0% | 3% | 51% | 77% | 29% | 29% | 60% | 14% | 6% | 9% | 9% | 0% |
| 2020 n=49 | 86% | 80% | 0% | 2% | 34% | 82% | 18% | 18% | 45% | 6% | 4% | 4% | 4% | 4% |
| 2021 n=44 | 98% | 95% | 0% | 7% | 36% | 91% | 39% | 39% | 61% | 7% | 7% | 5% | 7% | 0% |
| 2022 n=46 | 91% | 93% | 0% | 7% | 22% | 91% | 26% | 26% | 52% | 2% | 7% | 2% | 2% | 0% |
| 2023 n=26 | 88% | 88% | 0% | 4% | 15% | 88% | 23% | 23% | 27% | 12% | 0% | 0% | 4% | 4% |
| 2024 n=22 | 86% | 82% | 0% | 0% | 23% | 82% | 27% | 27% | 41% | 0% | 9% | 9% | 0% | 0% |
| 2025 n=1 | 100% | 100% | 0% | 0% | 0% | 100% | 0% | 0% | 100% | 0% | 0% | 0% | 0% | 0% |
